# Neutrophil-to-lymphocyte ratio is associated with sarcopenia risk in overweight maintenance hemodialysis patients

**DOI:** 10.1038/s41598-024-54056-2

**Published:** 2024-02-14

**Authors:** Huibin Nie, Yan Liu, Xiaoyan Zeng, Min Chen

**Affiliations:** 1 Department of Nephrology, Chengdu First People’s Hospital, Integrated TCM and Western Medicine Hospital Affiliated to Chengdu University of TCM, No. 18 Wanxiang North Street, Chengdu, 610095 Sichuan China; 2 Department of Clinical Nutrition, Chengdu First People’s Hospital, Integrated TCM and Western Medicine Hospital Affiliated to Chengdu University of TCM, No. 18 Wanxiang North Street, Chengdu, 610095 Sichuan China

**Keywords:** Risk factors, Haemodialysis

## Abstract

Neutrophil-to-lymphocyte ratio (NLR), a novel inflammatory marker, is strongly associated with the risk of sarcopenia. Notably, being overweight has been found to accelerate the loss of skeletal muscle mass and function in chronic kidney disease (CKD) patients. However, the effect of overweight status on the relationship between NLR and sarcopenia risk has been poorly studied. We conducted a cross-sectional study at a hemodialysis center in Chengdu, China, from September to December 2022. The prevalence of sarcopenia was determined according to the Asian Working Group for Sarcopenia (AWGS). Participants were stratified based on body mass index (BMI) categories for the Asian population (non-overweight < 23 kg/m^2^ and overweight ≥ 23 kg/m^2^). 272 participants aged 18–85 years were included, with 144 being male. The overall prevalence of sarcopenia was 32.72% (89/272). After adjusting for covariates, NLR was significantly associated with sarcopenia risk in overweight participants (OR 1.60, 95% CI 1.15–2.24, *p* = 0.006), whereas it was not significant in the non-overweight group (OR 0.88, 95% CI 0.70–1.10, *p* = 0.254). Moreover, subgroup analysis showed a significant interactive association between NLR and overweight status with respect to sarcopenia. These findings emphasize the potential significance of regular screening of NLR for the early detection of sarcopenia in overweight patients undergoing maintenance hemodialysis.

## Introduction

Sarcopenia, characterized by the progressive and systemic loss of muscle mass and function^1^, remains an underestimated concern in clinical practice, particularly within the context of chronic kidney disease (CKD)^2,3^. The prevalence of sarcopenia among patients with end-stage renal disease (ESRD) is 20–44%^4^ and approximately 50% in maintenance hemodialysis (MHD) patients^5,6^, which is much higher than that in the general population^7^. Moreover, overall quality of life is impaired in patients with sarcopenia. In patients undergoing MHD, the presence of sarcopenia is linked to an elevated risk of disability, and cardiovascular events^8^, along with a higher probability of rehospitalization and mortality^3,9^, which has been correlated with increased healthcare costs.

Multiple investigations have provided crucial evidences supporting the foundational role of chronic systemic inflammatory processes in the pathogenesis of CKD-associated sarcopenia^10,11^. Increased levels of circulating inflammatory markers are significantly associated with reduced skeletal muscle strength and muscle mass^12,13^. These inflammatory cytokines inhibit protein synthesis, reduce muscle anabolism and damage energy homeostasis, leading to muscle atrophy and malfunction^11^. The neutrophil-to-lymphocyte ratio (NLR), which is derived from absolute neutrophil and lymphocyte counts, is a convenient and cost-effective measure of systemic inflammation^14,15^. A previous study indicated that NLR could offer valuable insights into inflammation among CKD patients, including those undergoing hemodialysis^16^. Recent studies have assessed the correlation between NLR and the components of sarcopenia in different populations, including community dwelling older adults^17^ and patients with cancer or undergoing hemodialysis^13,18^. However, while some studies have shown significant associations between the NLR and sarcopenia risk^13,18, 19^, others have not^17,20^, which indicates the association between the NLR and sarcopenia risk remains uncertain.

Additionally, being overweight is linked to the accumulation of excess adipose tissue, a condition associated with chronic inflammation^21^. This, in turn, may exacerbate sarcopenia^22^, contributing to increased rates of disability, morbidity, and mortality. In obese individuals, visceral fat releases pro-inflammatory and chemotactic compounds, thereby rendering the muscle more susceptible to dysfunction, possibly through the inhibition of protein synthesis. Furthermore, fat deposition in skeletal muscles may result in local inflammation, leading to reduced muscle mass^23^. Among community-dwelling elderly women, individuals characterized by both obesity and low muscle mass exhibited a greater likelihood of having difficulties in physical function when compared to those with a healthier body composition^24^. Furthermore, evidence from observational studies suggests an association between being overweight and an elevated NLR^25^. Similarly, in ESRD patients, being overweight is also correlated with an inflammatory state^26,27^.

Given the association of subclinical inflammation with both overweight status and sarcopenia, it is plausible that the relationship between inflammation and sarcopenia may be influenced by overweight status. Despite extensive investigations into the association between NLR and the risk of sarcopenia, as well as the link between obesity and sarcopenia risk, studies related to the association between NLR, being overweight, and sarcopenia in patients undergoing MHD are limited. In the present study, we aimed to deepen the understanding of the relationship between inflammatory processes and sarcopenia in hemodialysis by exploring the relationship between NLR and the risk of sarcopenia in MHD patients with and without overweight.

## Results

### Characteristics of the study population

**Figure 1:**
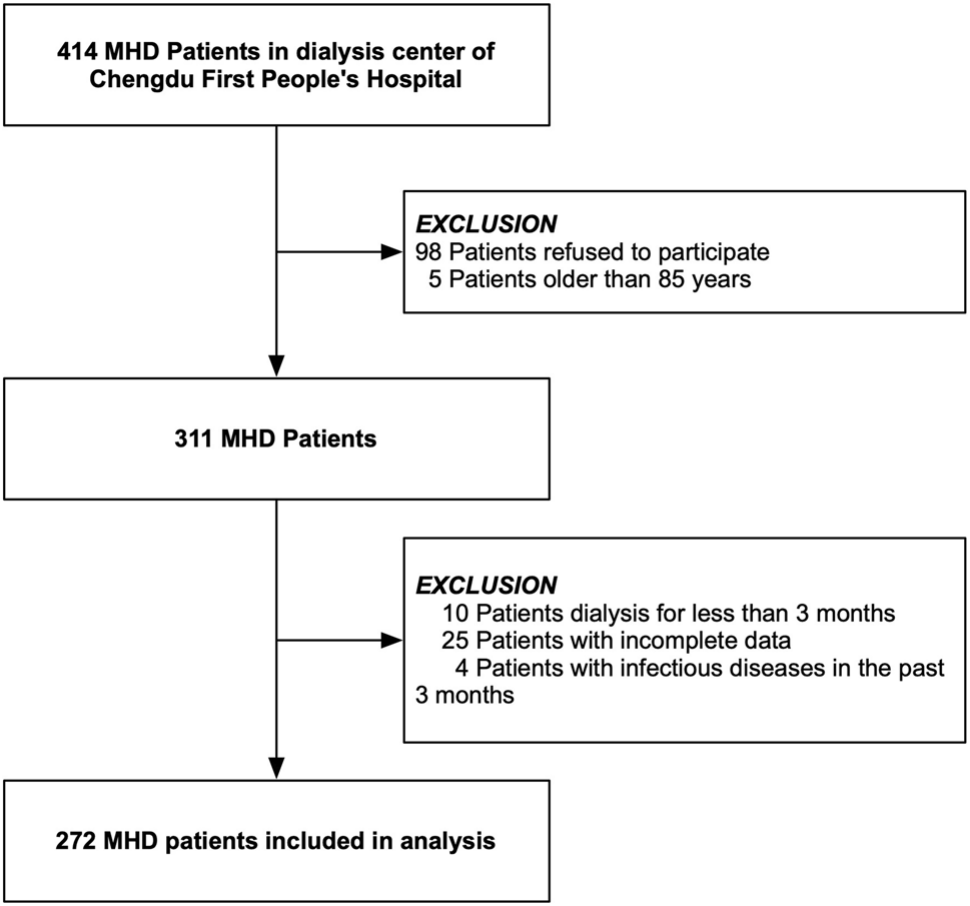
Schematic representation of the participant selection process and distribution of participant groups. *MHD* Maintenance hemodialysis.

From September to December 2022, a total of 272 participants were included in our analysis, as shown in Fig. 1. The demographic and clinical characteristics of the participants with and without sarcopenia are presented in Table 1. On average, the participants were 60 ± 13.9 years old, and 52.94% were men. The prevalence of sarcopenia was 32.72% (89/272). In comparison to the non-sarcopenia group, individuals with sarcopenia were older and had a higher MIS, age-adjusted CCI, and prevalence of diabetes. However, they had lower BMI and albumin levels.

**Table 1:** Comparisons of the characteristics between MHD patients with and without sarcopenia.

Variables	Total (n = 272)	Non-sarcopenia (n = 183)	Sarcopenia (n = 89)	*p* value
Demographic data				
Sex, n (%)				0.456
Male	144 (52.9)	94 (51.4)	50 (56.2)	
Female	128 (47.1)	89 (48.6)	39 (43.8)	
Age (years)	60.0 ± 13.9	56.2 ± 13.6	67.8 ± 11.2	< 0.001
Dialysis duration (months)	55.6 ± 44.8	56.7 ± 45.7	53.5 ± 43.0	0.587
Comorbidities				
T2D, n (%)	74 (27.2)	42 (23)	32 (36)	0.024
Age-adjusted CCI	4.9 ± 1.6	4.4 ± 1.5	5.8 ± 1.5	< 0.001
MIS	4.8 ± 3.0	4.0 ± 2.2	6.6 ± 3.6	< 0.001
Anthropometric characteristics				
BMI (kg/m^2^)	22.3 ± 3.8	23.1 ± 3.6	20.7 ± 3.4	< 0.001
ASMI (kg/m^2^)	6.5 ± 1.1	6.8 ± 1.0	5.8 ± 0.9	< 0.001
Fat mass (kg)	15.2 ± 7.6	16.0 ± 7.8	13.5 ± 6.8	0.010
Muscle strength				
Handgrip strength (kg)	23.6 ± 9.0	26.4 ± 8.7	17.9 ± 6.7	< 0.001
Functional capacity				
Gait speed (m/s)	0.8 ± 0.2	0.9 ± 0.2	0.6 ± 0.2	< 0.001
Laboratory parameters				
Hemoglobin (g/L)	107.8 ± 17.7	107.6 ± 16.7	108.1 ± 19.6	0.829
NLR	4.0 ± 1.9	3.9 ± 1.6	4.3 ± 2.4	0.105
HLDL-c (mmol/L)	0.9 ± 0.3	1.0 ± 0.3	0.9 ± 0.3	0.553
LLDL-c (mmol/L)	2.3 ± 0.7	2.3 ± 0.7	2.2 ± 0.8	0.238
TG (mmol/L)	1.9 (1.3, 2.8)	2.1 (1.4, 3.0)	1.7 (1.2, 2.6)	0.056
TC (mmol/L)	3.7 ± 0.9	3.7 ± 0.9	3.6 ± 1.0	0.129
Albumin (g/L)	39.2 ± 3.6	40.0 ± 3.2	37.6 ± 3.8	< 0.001
BUN (mmol/L)	21.3 ± 7.8	21.9 ± 8.2	20.2 ± 6.9	0.095
Creatinine (μmol/L)	871.6 ± 268.2	937.6 ± 263.5	735.8 ± 223.9	< 0.001
Uric acid (μmol/L)	404.6 ± 113.9	411.8 ± 113.4	389.8 ± 114.1	0.134
P (mmol/L)	1.6 ± 0.5	1.7 ± 0.5	1.6 ± 0.5	0.203
Ca (mmol/L)*	2.6 ± 0.5	2.5 ± 0.4	2.8 ± 0.7	< 0.001
iPTH (pg/ml)	245.5 (149.1, 421.3)	268.9 (158.2, 437.1)	219.2 (144.9, 369.1)	0.059
AKP(U/L)	91.0 (75.0, 114.2)	91.0 (74.5, 111.0)	92.0 (76.0, 116.0)	0.929
β2 microglobulin (mg/dL)	42.6 ± 12.8	42.4 ± 13.0	43.1 ± 12.6	0.708
hs-CRP (mg/dL)	3.3 (1.4, 6.8)	2.9 (1.4, 6.2)	4.2 (1.8, 8.9)	0.034
spKt/V	1.5 ± 0.6	1.5 ± 0.7	1.4 ± 0.3	0.797

*OR* Odds ratio, *95% CI* 95% Confidence interval, *Age-adjusted CCI* Age-adjusted Charlson comorbidity index, *BMI* Body mass index, *T2D* Type 2 diabetes, *ASMI* Appendicular Skeletal Muscle Mass Index, *NLR* Neutrophil to lymphocyte ratio, *HLDL-c* High density lipoprotein cholesterol, *LLDL-c* Low density lipoprotein cholesterol, *TG* Triglyceride, *TC* Total cholesterol, *BUN* Blood urea nitrogen, *iPTH* Intact parathyroid hormone, *AKP* Alkaline phosphatase, *spKt/V* Single-pool urea clearance index, *hs-CRP* High-sensitivity C-reactive protein, *MIS* Malnutrition inflammation score.

*We modified serum Calcium with serum albumin.

### Associations between NLR and the risk of sarcopenia in overweight and non-overweight MHD patients

**Figure 2:**
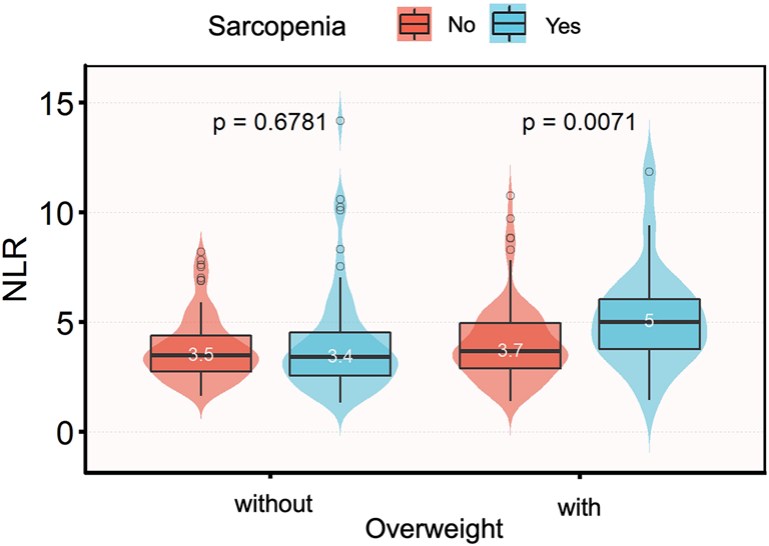
Distribution of NLR in MHD patients with sarcopenia grouped by overweight status (n = 272). NLR levels were compared between participants with and without sarcopenia in overweight or non-overweight MHD patients using the unpaired t-test separately. Boxplots express medians, interquartile ranges, and ranges. Circles indicate outliers. *NLR* Neutrophil to lymphocyte ratio.

Univariate analysis indicated that age, diabetes status, MIS, age-adjusted CCI, hs-CRP, and albumin were significantly associated with the risk of sarcopenia in both overweight and non-overweight participants. Notably, NLR exhibited a significant correlation with sarcopenia risk only in overweight MHD patients (Table 2). As shown in Fig. 2, within the overweight group, NLR was significantly higher in MHD patients with sarcopenia compared to those without sarcopenia (3.7 vs. 5.0, *p* = 0.007). Furthermore, as demonstrated in multivariate logistic regression analysis, a higher NLR was significantly associated with increased odds of sarcopenia (OR 1.60, 95% CI 1.15–2.24, *p* = 0.006) in overweight participants, after adjusting for previously mentioned confounders. However, this association was not significant in the non-overweight group (OR 0.88, 95% CI 0.70–1.10; *p* = 0.254) (Table 3). The significant relationship persisted in overweight MHD patients (*p* for trend = 0.006) but not in the non-overweight groups (*p* for trend = 0.168) when NLR was transformed into a categorical variable.

**Table 2:** Association of covariates and sarcopenia risk.

Variable	Overweight MHD patients (n = 113)		Non-overweight MHD patients (n = 159)	
	OR (95% CI)	*p* value	OR (95% CI)	*p* value
Demographic data				
Sex				
Male	1.00 (Ref)		1.00 (Ref)	0.462
Female	0.697 (0.266–1.823)	0.462	0.789 (0.42–1.483)	
Age (years)	1.127 (1.062–1.196)	< 0.001	1.08 (1.047–1.113)	< 0.001
Dialysis duration (months)	1.005 (0.993–1.017)	0.393	0.994 (0.988–1.001)	0.096
Comorbidities				
T2D				
No	1.00 (Ref)		1.00 (Ref)	0.004
Yes	1.935 (0.755–4.962)	0.169	3.362 (1.489–7.589)	
Age-adjusted CCI	2.363 (1.578–3.538)	< 0.001	1.437 (1.242–1.662)	< 0.001
MIS	1.249 (1.032–1.511)	0.022	2.107 (1.597–2.779)	< 0.001
Anthropometric characteristics				
Fat mass (kg)	1.011 (0.935–1.093)	0.784	1.013 (0.946–1.086)	0.703
Laboratory parameters				
Hemoglobin (g/L)	0.985 (0.962–1.009)	0.224	1.007 (0.988–1.026)	0.473
NLR	1.317 (1.051–1.65)	0.017	1.067 (0.905–1.258)	0.443
Albumin (g/L)	0.87 (0.762–0.994)	0.041	0.766 (0.683–0.859)	< 0.001
BUN (mmol/L)	0.948 (0.882–1.019)	0.144	0.97 (0.925–1.016)	0.197
Creatinine (μmol/L)	0.997 (0.995–0.999)	0.006	0.996 (0.994–0.997)	< 0.001
Uric acid (μmol/L)	0.998 (0.995–1.002)	0.412	0.998 (0.995–1.001)	0.260
HLDL-c (mmol/L)	0.559 (0.08–3.888)	0.556	0.35 (0.107–1.143)	0.082
LLDL-c (mmol/L)	0.844 (0.43–1.655)	0.621	0.734 (0.479–1.126)	0.157
TG (mmol/L)	0.764 (0.514–1.137)	0.185	1.021 (0.861–1.211)	0.810
TC (mmol/L)	0.761 (0.442–1.311)	0.325	0.769 (0.549–1.077)	0.127
P (mmol/L)	0.698 (0.265–1.835)	0.466	0.773 (0.419–1.427)	0.411
Ca (mmol/L)*	1.978 (0.831–4.706)	0.123	4.63 (2.120–10.113)	< 0.001
iPTH (pg/ml)	0.998 (0.995–1.001)	0.115	0.999 (0.998–1.000)	0.263
AKP (U/L)	0.996 (0.984–1.009)	0.543	0.999 (0.996–1.002)	0.496
β2 microglobulin (mg/dL)	1.023 (0.982–1.066)	0.268	0.993 (0.970–1.017)	0.582
hs-CRP (mg/dL)	1.025 (1.001–1.049)	0.040	1.09 (1.017–1.170)	0.016
spKt/V	0.993 (0.386–2.552)	0.988	0.689 (0.349–1.359)	0.282

*OR* Odds ratio, *95% CI* 95% Confidence interval, *Age-adjusted CCI* Age-adjusted Charlson comorbidity index, *BMI* Body mass index, *T2D* Type 2 diabetes, *ASMI* Appendicular Skeletal Muscle Mass Index, *NLR* Neutrophil to lymphocyte ratio, *HLDL-c* High density lipoprotein cholesterol, *LLDL-c* Low density lipoprotein cholesterol, *TG* Triglyceride, *TC* Total cholesterol, *BUN* Blood urea nitrogen, *iPTH* Intact parathyroid hormone, *AKP* Alkaline phosphatase, *spKt/V* Single-pool urea clearance index, *hs-CRP* High-sensitivity C-reactive protein, *MIS* Malnutrition inflammation score.

*We modified serum Calcium with serum albumin.

**Table 3:** The associations between NLR and the risk of sarcopenia in patients on MHD by overweight status.

Variables	Overweight MHD patients (n = 113)				Non-overweight MHD patients (n = 159)			
	Model I		Model II		Model I		Model II	
	OR (95% CI)	*p* value	OR (95% CI)	*p* value	OR (95% CI)	*p* value	OR (95% CI)	*p* value
NLR	1.32 (1.05–1.65)	0.017	1.60 (1.15–2.24)	0.006	1.07 (0.9–1.26)	0.443	0.88 (0.70–1.10)	0.254
NLR tertiles								
T1 < 3.12	1.00 (Ref)		1.00 (Ref)		1.00 (Ref)		1.00 (Ref)	
T2 ≥ 3.12 to < 4.26	1.61 (0.35–7.36)	0.538	1.71 (0.25–11.58)	0.581	0.73 (0.34–1.54)	0.403	0.47 (0.17–1.31)	0.149
T3 ≥ 4.26	4.23 (1.1–16.22)	0.036	10.13 (1.60–64.20)	0.014	1.11 (0.51–2.42)	0.792	0.51 (0.18–1.43)	0.203
*p* for trend		0.021		0.006		0.857		0.168

Model I: adjust for none.

Model II: Adjust for age, sex, MIS, albumin, hs-CRP, BMI and serum modified Calcium.

*OR* Odds ratio, *CI* Confidence interval, *Ref* Reference, *BMI* Body mass index, *hs-CRP* Hypersensitive C-reactive protein, *MIS* Malnutrition inflammation score.

Moreover, an interaction was found between overweight status and the NLR with respect to sarcopenia (*p* value for interaction = 0.011). Furthermore, even after translating NLR into a categorical variable, the interaction remained statistically significant (*p* value for interaction = 0.028) (Table 4).

**Table 4:** Interactive effect of NLR and risk of sarcopenia in MHD patients with or without overweight.

Variables	Non-overweight (n = 159)		Overweight (n = 113)		*p* for interaction
	OR (95% CI)	*p* value	OR (95% CI)	*p* value	
NLR	0.88 (0.70–1.10)	0.254	1.60 (1.15–2.24)	0.006	0.011
NLR tertiles					0.028
T1 (< 3.12)	1.00 (Ref)		1.00 (Ref)		
T2 (≥ 3.12 to < 4.26)	0.47 (0.17–1.31)	0.149	1.71 (0.25–11.58)	0.581	
T3 (≥ 4.26)	0.51 (0.18–1.43)	0.203	10.13 (1.6–64.20)	0.014	
Trend test		0.168		0.006	

Adjusted for age, sex, malnutrition inflammation score, albumin, hs-CRP and serum modified Calcium.

*NLR* Neutrophil to lymphocyte ratio, *OR* Odds ratio, *95% CI* 95% Confidence interval.

### Sensitivity analysis

Sensitivity analyses were conducted to confirm these conclusions. The results remained stable after applying the Working Group of Obesity in China (WGOC) criteria for overweight diagnosis. In the overweight group, the risk of sarcopenia increased significantly with a rising NLR (OR 1.61; 95% CI 1.11–2.34, *p* = 0.012), whereas the association was not significant in the non-overweight group (OR 0.90; 95% CI 0.72–1.13, *p* = 0.361) (Supplementary Table S1 online). There was an interaction between NLR and sarcopenia risk in MHD patients with overweight and those without overweight. (*p* value for interaction = 0.023).

The relationship between NLR and sarcopenic obesity risk was also performed as part of the sensitivity analyses. The characteristics of MHD patients with sarcopenic obesity versus non-obese sarcopenia are list in Supplementary Table S2. Overall, the average age of participants was 67.8 ± 11.2 years, and 56.2% were men. Compared with the non-obese sarcopenia group, individuals with sarcopenic obesity exhibited higher BMI, fat mass and NLR, but lower hemoglobin and spKt/V. Univariate analysis indicated that NLR, spKt/V and HLDL-c were significantly associated with the risk of sarcopenic obesity (Supplementary Table S3 online). Moreover, after adjusting for potential confounders, including age, sex and spKt/V, higher NLR remained significantly associated with increased odds of sarcopenic obesity (OR 1.40, 95% CI 1.11–1.40, *p* = 0.005) (Supplementary Table S4 online).

## Discussion

In this cross-sectional study involving 272 patients undergoing maintenance hemodialysis, we investigated the correlation between the NLR and the risk of sarcopenia. Additionally, we explored the potential modification of this relationship by overweight status. The findings revealed a significant association between NLR and sarcopenia risk among overweight MHD patients, even after adjusting for potential covariates. Conversely, this association was not observed in the non-overweight group. Subgroup analysis further indicated that the interaction of NLR with overweight status on the risk of sarcopenia was significant. This suggests that overweight status plays a crucial role as a modifier in the relationship between NLR and sarcopenia risk.

Numerous studies have shown that the NLR is a convenient and cost-effective marker of inflammation^28^ and an indicator of sarcopenia risk^15^. Previous research has evaluated the correlation between NLR and the risk of sarcopenia in different populations. For instance, a retrospective study involving 343 Chinese patients with renal cell carcinoma found an association between sarcopenia risk and NLR^18^, which was in line with the findings of Borges et al. and Liu et al.^29,30^. These studies suggested that a higher NLR might contribute to an elevated risk of sarcopenia in patients with hematological or solid cancers. Additionally, Wang et al. discovered a negative correlation between NLR and grip strength, gait speed, and skeletal muscle mass index in patients undergoing MHD^13^. In accordance with these studies, our research provided robust evidence supporting an association between NLR and the risk of sarcopenia in overweight patients undergoing MHD. Conversely, in non-overweight participants, the relationship between NLR and sarcopenia risk was not statistically significant. This pattern parallels findings in kidney transplantation patients (KTPs), where NLR was not significantly correlated with muscle mass, strength, or functional capacity^20^. Tang et al. also reported a lack of association between NLR and appendicular skeletal muscle or handgrip strength^17^ in Chinese community-dwelling older people.

Why is the association between NLR and sarcopenia risk so different in different populations? This may due to the difference in systemic inflammation status influenced by different overweight status, which has not been previously explored in depth^17^. Overweight is the result of chronic energy imbalance, and excess energy is stored in the form of adipose tissue, leading to an elevation of chronic inflammation^21^. Intermuscular adipose tissue (IMAT) and intramyocellular lipids (MCLs), induced by fat deposition in skeletal muscles, are in close proximity to muscle fibers. They release a cascade of pro-inflammatory cytokines into muscle fibers, thereby resulting in local inflammation^23,31, 32^. Moreover, in adipose tissue (AT), adipocytes undergo hypertrophy, hyperplasia, and activation, leading to the infiltration of macrophages and other immune cells and dysregulated production of pro-inflammatory cytokines and chemokines. These processes contribute to both local and systemic chronic inflammation and therefore render muscle fibers more vulnerable to sarcopenia, potentially by triggering protein degradation, mitochondrial dysregulation, and insulin resistance^32^. Similarly, in a cohort study involving 328 patients with ESRD, Honda et al. found that adipose tissue in overweight patients was a source of pro-inflammatory mediators. The highest concentrations of these mediators were observed in overweight patients and were significantly associated with protein-energy wasting (PEW), a crucial contributor to sarcopenia^26^. In accordance with previous results, our study revealed that overweight MHD patients had higher levels of fat mass and inflammatory factors compared with non-overweight patients. Notably, the risk of sarcopenia exhibited a significant increase with rising NLR in overweight patients undergoing MHD, in contrast to non-overweight MHD patients. It can be assumed that in participants who had relatively low-grade inflammation, there was no apparent correlation between NLR and the risk of sarcopenia, aligning with the results reported by Ho et al.^20^. Conversely, in individuals with higher-grade inflammation, an elevated NLR is indicative of an increased risk of sarcopenia. Furthermore, our investigation revealed that in a special synergistic complication arising from both sarcopenia and obesity, known as sarcopenic obesity^33^, the NLR was higher and significantly associated with an elevated risk of sarcopenic obesity in comparison to non-obese sarcopenia MHD patients. These findings highlight the significance of overweight status as a crucial modifier in the relationship between NLR and the risk of sarcopenia in participants undergoing maintenance hemodialysis. It is recommended that routine screening of NLR levels be considered essential for the early detection of sarcopenia in overweight MHD patients.

This study has several strengths. Firstly, despite extensive investigations into the association between NLR and the risk of sarcopenia across various populations, the results have remained controversial^13,20^. Notably, the role of overweight in both inflammation and sarcopenia development has been underscored, yet few studies have explored the interaction between overweight status and NLR concerning sarcopenia. Our study, which examined the impact of overweight status, revealed a significant interaction effect between overweight status and the association of NLR with the risk of sarcopenia. Furthermore, our findings not only contribute to the existing body of knowledge but also suggest a promising direction for future trials that aim to delve deeper into the intricate relationship of NLR and overweight status with the occurrence of sarcopenia in chronic kidney disease (CKD) patients. In addition, our analyses were adjusted for several crucial confounders of nutritional and inflammatory status, including serum calcium level, MIS, and hs-CRP level, all of which are relevant to the prognosis of sarcopenia. This comprehensive adjustment strategy enhances the reliability of our results.

Nevertheless, this study has some limitations. Firstly, since this is a cross-sectional study, the causal association of NLR with the development of sarcopenia in overweight MHD patients cannot be clearly elucidated. To address this limitation, we plan to undertake a cohort study based on the present data to comprehensively assess the associations among overweight status, inflammation markers, and sarcopenia. Secondly, it's essential to recognize that body weight and ASM may be influenced by the hydration state of patients undergoing MHD. In an effort to minimize the impact of fluid overload, we assessed ASM using a bioelectrical impedance analyzer after a midweek, adequately conducted hemodialysis session—a methodology consistent with previous studies^34,35^. Furthermore, in previous investigations employing serial assessments of ASM using multiple-frequency bioimpedance in peritoneal dialysis patients, there was no correlation between changes in hydration status and observed lean body mass alterations over time^36^.

In conclusion, our study revealed that the association between neutrophil-to-lymphocyte ratio and sarcopenia risk was influenced by the overweight status of MHD patients. Specifically, NLR exhibited a significant association with an elevated risk of sarcopenia in overweight MHD patients, whereas this association was not significant in the non-overweight group. These findings underscore the importance of NLR as an accessible and indirect indicator of sarcopenia risk in overweight individuals undergoing maintenance hemodialysis. Further research should identify NLR reference ranges in overweight and non-overweight MHD populations to improve their applicability in clinical practice. Additionally, there is a pressing need to investigate the potential role of NLR in stratifying sarcopenia risk among MHD patients.

## Methods

### Study design and participants

This cross-sectional study included patients aged 18–85 years with ESRD undergoing maintenance hemodialysis at Chengdu First People's Hospital from September 2022 to December 2022. Patients were excluded under the following conditions: refusal to participate (n = 98), dialysis duration less than 3 months (n = 10), history of infectious disease within the past 3 months (n = 4), inability to complete the physical test due to cerebrovascular or severe cardiopulmonary disease (n = 12), or absence of a blood test (n = 13). Thus, 272 participants were included in the analysis (Fig. 1). All enrolled participants underwent hemodialysis three times a week (4 h per session) using bicarbonate dialysate and cellulose acetate, or polysulfone dialysis membranes. The majority of patients (95%) were prescribed antihypertensive medications and other standard treatments for ESRD, such as phosphate binders, erythropoietin, and vitamin D supplements.

### Ethics statement

This study was in accordance with the principles of the Declaration of Helsinki and was approved by the Ethics Committee of Chengdu First People's Hospital (approval number: 2022KT003). All the participants provided written informed consent to participate in the study.

### Definitions of NLR, sarcopenia and overweight

NLR was calculated as the absolute neutrophil counts divided by the absolute lymphocyte counts.

Sarcopenia was defined in accordance with the 2019 consensus of the Asian Working Group for Sarcopenia (AWGS)^37^. This definition encompasses criteria including low muscle strength (handgrip strength: < 28 kg for men, < 18 kg for women), low muscle mass (appendicular skeletal muscle mass index (ASMI) assessed by bioelectrical impedance analysis (BIA): < 7.0 kg/m^2^ for men, 5.7 kg/m^2^ for women), and impaired physical performance determined by a 6-m walk < 1.0 m/s. Sarcopenia was defined as the presence of both low muscle mass and strength (or low physical performance).

Handgrip strength was measured using an electronic grip strength meter (EH101, Xiangshan Inc., Guangdong, China) before midweek hemodialysis. Patients were required to hold the grip strength meter perpendicularly with the elbow by the side of the body and handle it appropriately to guarantee maximal voluntary exertion. Three measurements of the arm without a fistula were performed, and the highest recorded value was documented. The gait speed test was performed before hemodialysis without any assistance. Each patient completed two 6-m walks at a regular pace, and the average of two consecutive measurements was documented. Appendicular skeletal muscle mass (ASM) was measured using a bioelectrical impedance analyzer (Inbody S10, BioSpace, Seoul, Korea) 20–30 min after the midweek hemodialysis session^38^, and the ASMI was calculated as ASM (kg)/height^2^ (m^2^).

Overweight was defined using the World Health Organization classification for the Asian population^39^ and participants were classified into two groups according to body mass index (BMI): non-overweight (BMI < 23 kg/m^2^) and overweight (≥ 23.0 kg/m^2^), respectively. BMI was defined as weight (dry weight) in kilograms divided by the square of the height in meters.

### Covariates

Demographic information, including age, sex, and dialysis duration, was obtained from medical records. Concurrent chronic conditions, such as chronic obstructive pulmonary disease, hypertension, coronary heart disease, diabetes mellitus, arthritis, stroke, and tumors, were self-reported and corroborated with medical records. The age-adjusted Charlson comorbidity index (CCI) was calculated based on the gathered data^40^.

Laboratory examination data were measured before a midweek pre-dialysis session, including neutrophil and lymphocyte counts, hemoglobin, plasma lipids, albumin, alkaline phosphatase, creatinine, uric acid, urea nitrogen, serum calcium, serum phosphorus, intact parathyroid hormone(iPTH), high-sensitivity C-reactive protein(hs-CRP), and β2 microglobulin. The albumin-corrected calcium level was calculated as serum calcium + (40-albumin(g/L)) × 0.02. Additionally, the single-pool urea clearance index (spKt/V) was calculated using pre- and post-dialysis urea nitrogen measurements, applying the formula described by Daugirdas et al.

Nutritional status was evaluated by calculating the malnutrition inflammation score (MIS) as previously indicated^41^. The assessment comprises four sections: medical history (including changes in dry weight, dietary intake, gastrointestinal symptoms, functional capacity, and comorbidities), physical examination, BMI, and laboratory tests. Each section was evaluated on a four-point severity scale, ranging from 0 (normal) to 3 (very severe). The cumulative score of all 10 components produced an overall MIS ranging from 0 (normal) to 30 (severely malnourished).

### Statistical analysis

Participant characteristics were summarized as mean (standard deviation [SD]) for continuous variables with a normal distribution or as median (interquartile range [IQR]) for a skewed distribution. The t-test was employed for analyzing normally distributed continuous variables, while the Mann–Whitney U test was utilized for skewed distribution. Categorical variables were presented as percentages and compared using the chi-square test or Fisher probabilities.

Univariate and multivariate logistic regression analyses were used to investigate the relationship between NLR and the risk of sarcopenia. In all analyses, the percentages of missing values of covariates were below 3%. Specifically, four participants (1.47%) had missing values for plasma lipids and one (0.36%) for hs-CRP or iPTH. Consequently, participants with relevant missing values were excluded. Variables either reported in prior study^17^ or assumed clinically relevant were included in the multivariable logistic regression analysis model. Additionally, covariates were integrated into the final models as potential confounders if they induced changes in the estimates of the NLR for sarcopenia by more than 10%.

Therefore, in the multivariate logistic regression, NLR was examined both as a continuous and categorical variable. For the categorical variable, NLR levels were divided into tertiles, with the subjects in the lowest tertile group serving as the reference. The cut-off levels for NLR tertiles were as follows: T1 < 3.12, T2 3.12 to < 4.26, and T3 ≥ 4.26. Model 1 was unadjusted, and Model 2 was adjusted for age, sex, MIS, albumin, BMI, hs-CRP and serum modified calcium. Subgroup analyses were performed between the non-overweight and overweight MHD groups. Interactions among the subgroups were analyzed using the likelihood ratio test.

Sensitivity analyses were performed by changing variable definitions in this study to confirm the stability of the findings^42^. An alternative definition for overweight was applied, following the criteria set by the Working Group of Obesity in China (WGOC) (non-overweight (BMI < 24 kg/m^2^) and overweight (≥ 24.0 kg/m^2^)).A distinct definition for a specific type of sarcopenia associated with overweight, known as sarcopenic obesity^33^, was also employed. As part of the sensitivity analysis, the association between NLR and sarcopenic obesity risk was assessed.

All of the analyses were performed with the statistical software packages R (http://www.R-project.org, The R Foundation) and Free Statistics software versions 1.7. All statistical tests were conducted as two-tailed and *p* value < 0.05 indicated statistical significance.

### Supplementary Information

Supplementary Information.

## Data Availability

The data analyzed in this study are available from the corresponding author upon reasonable request.
