# Supplementary material for: Neutrophil-to-lymphocyte ratio is associated with sarcopenia risk in overweight maintenance hemodialysis patients
Source: Sci Rep. 2024 Feb 14;14:3669. doi: 10.1038/s41598-024-54056-2 (PMC10864262; doi:10.1038/s41598-024-54056-2)
Supplement: Supplementary file 1 — Supplementary Information. [file 41598_2024_54056_MOESM1_ESM.docx]

**Supplementary methods**

**Definitions of sarcopenic overweight**

The current criteria for sarcopenic obesity rely on individual definitions of both sarcopenia and obesity^1^. In this study, sarcopenic obesity is defined in accordance with previous descriptions^2^. Obesity is characterized as BMI≥25kg/m^2^ according to World Health Organization classification for the Asian population. Sarcopenic obesity is then identified when both obesity and sarcopenia are present.

**Supplementary tables**

Supplementary Table S1. The associations between NLR and risk of sarcopenia in patients on MHD by overweight status (n=272) (WGOC criteria)

| **Variables** | **Non-overweight(n=181)** | | **Overweight(n=91)** | | ***p* for interaction** |
| --- | --- | --- | --- | --- | --- |
|  | **OR (95%CI)** | ***p* value** | **OR (95%CI)** | ***p* value** |  |
| **NLR** | 0.90(0.72~1.13) | 0.361 | 1.61 (1.11~2.34) | 0.012 | 0.023 |

Adjusted for age, sex, malnutrition inflammation score, albumin, hs-CRP and serum modified Calcium.

Abbreviations：NLR, neutrophil to lymphocyte ratio; OR, odds ratio; 95% CI, 95% confidence interval; WGOC criteria, Working Group of Obesity in China.

Supplementary Table S2. The Characteristics of MHD patients with sarcopenic obesity versus non-obese sarcopenia(n=89)

| **Variables** | **Total**  **(n = 89)** | **non-obese sarcopenia**  **(n = 76)** | **sarcopenic**  **obesity**  **(n = 13)** | ***p* value** |
| --- | --- | --- | --- | --- |
| ***Demographic data*** |  |  |  |  |
| Sex, n (%) |  |  |  | 0.305 |
| Man | 50 (56.2) | 41 (53.9) | 9 (69.2) |  |
| Female | 39 (43.8) | 35 (46.1) | 4 (30.8) |  |
| Age(years) | 67.8 ± 11.2 | 66.9 ± 11.6 | 73.2 ± 6.6 | 0.061 |
| Dialysis duration(months) | 53.5 ± 43.0 | 51.5 ± 43.1 | 65.3 ± 42.7 | 0.287 |
| ***Comorbidities*** |  |  |  |  |
| T2D, n (%) | 32 (36.0) | 27 (35.5) | 5 (38.5) | 1 |
| Age-adjusted CCI | 5.8 ± 1.5 | 5.7 ± 1.5 | 6.5 ± 1.3 | 0.075 |
| MIS | 6.6 ± 3.6 | 6.8 ± 3.7 | 5.5 ± 2.8 | 0.266 |
| ***Anthropometric characteristics*** |  |  |  |  |
| BMI (kg/m^2^) | 20.7 ± 3.4 | 19.7 ± 2.8 | 26.2 ± 0.9 | < 0.001 |
| ASMI (kg/m^2^) | 5.8 ± 0.9 | 5.7 ± 0.9 | 6.3 ± 0.7 | 0.019 |
| Fat mass(kg) | 13.5 ± 6.8 | 11.7 ± 5.6 | 23.8 ± 3.6 | < 0.001 |
| ***Muscle strength*** |  |  |  |  |
| Handgrip strength (kg) | 17.9 ± 6.7 | 18.0 ± 6.5 | 17.0 ± 8.0 | 0.621 |
| ***Functional capacity*** |  |  |  |  |
| Gait speed (m/sec) | 0.6 ± 0.2 | 0.7 ± 0.2 | 0.5 ± 0.2 | 0.023 |
| ***Laboratory parameters*** |  |  |  |  |
| Hemoglobin (g/L) | 108.1 ± 19.6 | 109.8 ± 19.1 | 98.2 ± 20.2 | 0.047 |
| NLR | 4.3 ± 2.4 | 4.0 ± 2.3 | 6.2 ± 2.3 | 0.002 |
| HLDL-c (mmol/L) | 0.9 ± 0.3 | 1.0 ± 0.3 | 0.7 ± 0.2 | 0.008 |
| LLDL-c (mmol/L) | 2.2 ± 0.8 | 2.2 ± 0.8 | 1.9 ± 0.6 | 0.211 |
| TG (mmol/L) | 1.7 (1.2, 2.6) | 1.7 (1.2, 2.6) | 1.6 (1.2, 2.5) | 0.801 |
| TC (mmol/L) | 3.6 ± 1.0 | 3.6 ± 1.0 | 3.1 ± 0.7 | 0.063 |
| Albumin(g/L) | 37.6 ± 3.8 | 37.5 ± 3.9 | 38.0 ± 3.1 | 0.614 |
| BUN (mmol/L) | 20.2 ± 6.9 | 20.6 ± 7.0 | 18.1 ± 6.2 | 0.234 |
| Creatinine (μmol/L) | 735.8 ± 223.9 | 741.5 ± 228.0 | 702.4 ± 203.0 | 0.563 |
| Uric acid (μmol/L) | 389.8 ± 114.1 | 391.9 ± 111.5 | 377.2 ± 132.6 | 0.668 |
| P(mmol/L) | 1.6 ± 0.5 | 1.6 ± 0.5 | 1.6 ± 0.2 | 0.634 |
| Ca(mmol/L) * | 2.8 ± 0.7 | 2.8 ± 0.7 | 2.7 ± 0.6 | 0.410 |
| iPTH (pg/ml) | 219.2(144.9,369.1) | 221.4(136.9,370.7) | 215.3(207.4,323.2) | 0.727 |
| AKP(U/L) | 92.0(76.0,116.0) | 92.5 (75.8, 116.2) | 89.0(81.0, 105.0) | 0.803 |
| β2 microglobulin (mg/dL) | 43.1 ± 12.6 | 42.6 ± 12.7 | 46.1 ± 12.1 | 0.366 |
| hs-CRP (mg/dL) | 4.2 (1.8, 8.9) | 3.6 (1.7, 8.6) | 5.7 (3.4, 12.8) | 0.148 |
| spKt/V | 1.4 ± 0.3 | 1.5 ± 0.3 | 1.2 ± 0.4 | 0.017 |

**Abbreviations:** OR, odds ratio; 95% CI, 95% confidence interval; Age-adjusted CCI, age-adjusted Charlson comorbidity index; BMI, body mass index; T2D, type 2 diabetes; ASMI, Appendicular Skeletal Muscle Mass Index; NLR, neutrophil to lymphocyte ratio; HLDL-c, high density lipoprotein cholesterol; LLDL-c, low density lipoprotein cholesterol; TG, Triglyceride; TC, total cholesterol; BUN, blood urea nitrogen; iPTH, intact parathyroid hormone; AKP, alkaline phosphatase; spKt/V, single-pool urea clearance index; hs-CRP, high-sensitivity C-reactive protein; MIS, malnutrition inflammation score.

*We modified serum Calcium with serum albumin.

Supplementary Table S3. Association of covariates and sarcopenic obesity risk (n=89)

| **Variable** |  | **OR（95%CI）** | ***p* value** |  |
| --- | --- | --- | --- | --- |
| ***Demographic data*** |  |  |  |  |
| Sex | Male | 1.00.(Ref) | 0.310 |  |
|  | Female | 0.521 (0.148~1.838) |  |  |
| Age(years) |  | 1.064 (0.996~1.138) | 0.066 |  |
| Dialysis duration (months) |  | 1.007 (0.994~1.02) | 0.287 |  |
| ***Comorbidities*** |  |  |  |  |
| T2D | NO | 1.00.(Ref) | 0.839 |  |
|  | YES | 1.134 (0.338~3.812) |  |  |
| Age-adjusted CCI |  | 1.531 (0.948~2.471) | 0.082 |  |
| MIS |  | 0.892 (0.73~1.09) | 0.263 |  |
| ***Laboratory parameters*** |  |  |  |  |
| Hemoglobin (g/L) |  | 0.968 (0.936~1) | 0.052 |  |
| NLR |  | 1.36 (1.09~1.697) | 0.006 |  |
| Albumin(g/L) |  | 1.044 (0.885~1.23) | 0.610 |  |
| HLDL-c (mmol/L) |  | 0.019 (0.001~0.412) | 0.012 |  |
| LLDL-c (mmol/L) |  | 0.547 (0.212~1.414) | 0.213 |  |
| TG (mmol/L) |  | 0.924 (0.606~1.409) | 0.714 |  |
| TC (mmol/L) |  | 0.461 (0.199~1.067) | 0.071 |  |
| P(mmol/L) |  | 1.341 (0.407~4.415) | 0.629 |  |
| Ca(mmol/L) * |  | 0.641 (0.224~1.836) | 0.408 |  |
| iPTH (pg/ml) |  | 0.999 (0.997~1.002) | 0.701 |  |
| AKP(U/L) |  | 0.997 (0.985~1.01) | 0.689 |  |
| β2 microglobulin (mg/dL) |  | 1.022 (0.975~1.072) | 0.363 |  |
| hs-CRP (mg/dL) |  | 1.006 (0.99~1.023) | 0.449 |  |
| spKt/V | |  | 0.172 (0.035~0.85) | 0.031 |

**Abbreviations:** OR, odds ratio; 95% CI, 95% confidence interval; Age-adjusted CCI, age-adjusted Charlson comorbidity index; T2D, type 2 diabetes; NLR, neutrophil to lymphocyte ratio; HLDL-c, high density lipoprotein cholesterol; LLDL-c, low density lipoprotein cholesterol; TG, Triglyceride; TC, total cholesterol; iPTH, intact parathyroid hormone; AKP, alkaline phosphatase; spKt/V, single-pool urea clearance index; hs-CRP, high-sensitivity C-reactive protein; MIS, malnutrition inflammation score.

*We modified serum Calcium with serum albumin.

Supplementary Table S4. Association between NLR and sarcopenic obesity in multivariate logistic regression model among MHD patients (n=89)

| **Variables** | **Non-adjusted Model** | | **Model I** | | **Model II** | |
| --- | --- | --- | --- | --- | --- | --- |
|  | **OR (95% CI)** | ***p* value** | **OR (95% CI)** | ***p* value** | **OR (95% CI)** | ***p* value** |
| **NLR** | 1.36 (1.09~1.70) | 0.006 | 1.37 (1.08~1.74) | 0.010 | 1.40 (1.11~1.40) | 0.005 |

**Abbreviations**: OR, odds ratio; CI, confidence interval; spKt/V, single-pool urea clearance index.

**Note:** Non-adjusted model: adjust for none.

Model I: Adjust for age and sex.

Model II: Adjust for age, sex, and spKt/V.

**Supplementary Reference**

1 Batsis, J. A. & Villareal, D. T. Sarcopenic obesity in older adults: aetiology, epidemiology and treatment strategies. *Nat Rev Endocrinol* **14**, 513-537, doi:10.1038/s41574-018-0062-9 (2018).

2 Jia, S. *et al.* Sex differences in the association of physical activity levels and vitamin D with obesity, sarcopenia, and sarcopenic obesity: a cross-sectional study. *BMC Geriatr* **22**, 898, doi:10.1186/s12877-022-03577-4 (2022).
